# 5-[(*E*)-Benzyl­idene]-2-hy­droxy-8,9-di­phenyl-3,10-diaza­hexa­cyclo­[10.7.1.1^3,7^.0^2,11^.0^7,11^.0^16,20^]henicosa-1(19),12(20),13,15,17-pentaen-6-one

**DOI:** 10.1107/S1600536811040645

**Published:** 2011-10-08

**Authors:** Raju Suresh Kumar, Hasnah Osman, Yalda Kia, Mohd Mustaqim Rosli, Hoong-Kun Fun

**Affiliations:** a School of Chemical Sciences, Universiti Sains Malaysia, 11800 USM, Penang, Malaysia; b X-ray Crystallography Unit, School of Physics, Universiti Sains Malaysia, 11800 USM, Penang, Malaysia

## Abstract

In the title compound, C_38_H_30_N_2_O_2_, the acenaphthyl­ene ring is close to being planar [maximum deviation = 0.1047 (11) Å]. The dihedral angles between the three benzene rings and the acenaphthyl­ene system are 39.47 (3), 37.65 (3) and 44.47 (3)°. An intra­molecular O—H⋯N inter­action forms an *S*(5) hydrogen-bond ring motif. In the crystal, mol­ecules are linked into [101] chains by a set of C—H⋯O inter­actions.

## Related literature

For background to synthetic routes to pyrrolidines, see: Lown (1984 ▶); Tsuge & Kanemasa (1989 ▶); Monlineux (1987 ▶); Hensler *et al.* (2006 ▶). For hydrogen-bond motifs, see: Bernstein *et al.* (1995 ▶). For stability of the temperature controller used in the data collection, see: Cosier & Glazer (1986 ▶).
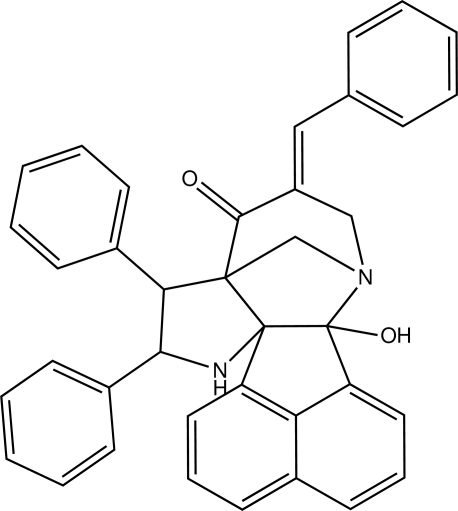


## Experimental

### 

#### Crystal data

C_38_H_30_N_2_O_2_
*M* *_r_* = 546.64
Triclinic, 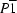

*a* = 9.0811 (1) Å
*b* = 11.7300 (1) Å
*c* = 14.0859 (2) Å
α = 75.828 (1)°
β = 75.470 (1)°
γ = 77.635 (1)°
*V* = 1389.49 (3) Å^3^
*Z* = 2
Mo *K*α radiation
μ = 0.08 mm^−1^
*T* = 100 K
0.38 × 0.34 × 0.28 mm

#### Data collection

Bruker SMART APEXII CCD diffractometer
Absorption correction: multi-scan (*SADABS*; Bruker, 2009) ▶ *T*_min_ = 0.970, *T*_max_ = 0.978
29369 measured reflections
7966 independent reflections
6687 reflections with *I* > 2σ(*I*)
*R*_int_ = 0.022

#### Refinement

*R*[*F* ^2^ > 2σ(*F* ^2^)] = 0.045
*wR*(*F* ^2^) = 0.120
*S* = 1.04
7966 reflections
387 parameters
H atoms treated by a mixture of independent and constrained refinement
Δρ_max_ = 0.44 e Å^−3^
Δρ_min_ = −0.22 e Å^−3^

### 

Data collection: *APEX2* (Bruker, 2009) ▶; cell refinement: *SAINT* (Bruker, 2009) ▶; data reduction: *SAINT*; program(s) used to solve structure: *SHELXTL* (Sheldrick, 2008 ▶); program(s) used to refine structure: *SHELXTL*; molecular graphics: *SHELXTL*; software used to prepare material for publication: *SHELXTL* and *PLATON* (Spek, 2009 ▶).

## Supplementary Material

Crystal structure: contains datablock(s) I, global. DOI: 10.1107/S1600536811040645/hb6415sup1.cif

Structure factors: contains datablock(s) I. DOI: 10.1107/S1600536811040645/hb6415Isup2.hkl

Supplementary material file. DOI: 10.1107/S1600536811040645/hb6415Isup3.cml

Additional supplementary materials:  crystallographic information; 3D view; checkCIF report

## Figures and Tables

**Table 1:** Hydrogen-bond geometry (Å, °)

*D*—H⋯*A*	*D*—H	H⋯*A*	*D*⋯*A*	*D*—H⋯*A*
O2—H1*O*2⋯N1	0.93 (2)	1.91 (2)	2.6348 (14)	133.7 (18)
C1—H1*A*⋯O1^i^	0.95	2.48	3.3874 (17)	160
C11—H11*A*⋯O2^ii^	0.99	2.57	3.5621 (13)	175
C19—H19*A*⋯O2^ii^	0.95	2.46	3.4044 (14)	176
C20—H20*A*⋯O2^ii^	1.00	2.42	3.4090 (15)	172

Symmetry codes: (i) 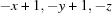; (ii) 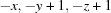.

**Fig. 1.**
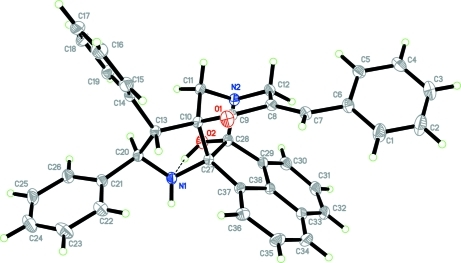
The molecular structure, showing 50% probability displacement ellipsoids. Hydrogen atoms are shown as spheres of arbitrary radius.

**Fig. 2.**
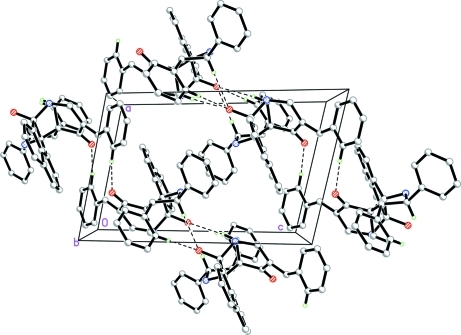
The crystal packing of (I). Dashed lines indicate hydrogen bonds. H atoms not involved in the hydrogen bond interactions have been omitted for clarity.

## supplementary crystallographic information

### Comment

Intermolecular 1,3-dipolar cycloadditions are considered as one of the most useful processes for the construction of five-membered rings containing the pyrrolidine structural unit (Lown *et al.*, 1984; Tsuge *et al.*, 1989). Functionalized pyrrolidine ring systems have acquired a prominent place among various heterocyclic compounds as it is the key structural motif in many pharmacologically relevant alkaloids (Monlineux, 1987). Recent drug developments incorporating the pyrrolidine motif have been identified as candidates with promising anti-HIV and antimicrobial activities (Hensler *et al.*, 2006). Due to the biological significance of the aforesaid heterocycle, the crystal structure determination of the title compound was carried out and the results are presented in this paper.

All parameters in (I) within normal ranges. The acenaphthylene ring (C27–C38) is almost planar with the maximum deviation of 0.1047 (11)Å for atom C27. It makes dihedral angles of 39.47 (3), 37.65 (3) and 44.47 (3)°, respectively, with the C1–C9, C14–C19 and C21–C26 benzene rings.

In the molecular structure, an intramolcular interaction was observed and form an S(5) hydrogen ring motif (Bernstein *et al.*, 1995). The crystal structure was arranged in the form of infinite chains along [101] by intermolecular C1—H1A···O1^i^, C11—H11A···O2^ii^, C19—H19A···O2^ii^ and C20—H20A···O2^ii^ interactions (Table 1).

### Experimental

A mixture of 3,5-bis[(*E*)-phenylmethylidene]tetrahydro-4(1*H*)-pyridinone (1 mmol), acenaphthenequinone (1 mmol), and phenylglycine (1 mmol) were dissolved in methanol (5 ml) and refluxed in a water bath for 1 h. After completion of the reaction as evident from TLC, the mixture was poured into water (50 ml). The precipitated solid was filtered and washed with water to obtain the product which was further purified by recrystallization from ethyl acetate to yield colourless blocks.

### Refinement

O and N bound H atoms were located from a difference Fourier maps and freely refined. The remaining H atoms were positioned geometrically and refined using a riding model with C—H = 0.95–1.00Å and *U*_iso_(H) = 1.2*U*_eq_(C).

### Crystal data

**Table:**

C_38_H_30_N_2_O_2_	*Z* = 2
*M**_r_* = 546.64	*F*(000) = 576
Triclinic, *P*1	*D*_x_ = 1.307 Mg m^−^^3^
Hall symbol: -P 1	Mo *K*α radiation, λ = 0.71073 Å
*a* = 9.0811 (1) Å	Cell parameters from 9941 reflections
*b* = 11.7300 (1) Å	θ = 2.4–29.9°
*c* = 14.0859 (2) Å	µ = 0.08 mm^−^^1^
α = 75.828 (1)°	*T* = 100 K
β = 75.470 (1)°	Block, colourless
γ = 77.635 (1)°	0.38 × 0.34 × 0.28 mm
*V* = 1389.49 (3) Å^3^	

### Data collection

**Table:**

Bruker SMART APEXII CCD diffractometer	7966 independent reflections
Radiation source: fine-focus sealed tube	6687 reflections with *I* > 2σ(*I*)
graphite	*R*_int_ = 0.022
φ and ω scans	θ_max_ = 29.9°, θ_min_ = 1.8°
Absorption correction: multi-scan (*SADABS*; Bruker, 2009)	*h* = −12→12
*T*_min_ = 0.970, *T*_max_ = 0.978	*k* = −16→16
29369 measured reflections	*l* = −19→13

### Refinement

**Table:**

Refinement on *F*^2^	Primary atom site location: structure-invariant direct methods
Least-squares matrix: full	Secondary atom site location: difference Fourier map
*R*[*F*^2^ > 2σ(*F*^2^)] = 0.045	Hydrogen site location: inferred from neighbouring sites
*wR*(*F*^2^) = 0.120	H atoms treated by a mixture of independent and constrained refinement
*S* = 1.04	*w* = 1/[σ^2^(*F*_o_^2^) + (0.0586*P*)^2^ + 0.5264*P*] where *P* = (*F*_o_^2^ + 2*F*_c_^2^)/3
7966 reflections	(Δ/σ)_max_ < 0.001
387 parameters	Δρ_max_ = 0.44 e Å^−^^3^
0 restraints	Δρ_min_ = −0.22 e Å^−^^3^

### Special details

**Table:**

Experimental. The crystal was placed in the cold stream of an Oxford Cryosystems Cobra open-flow nitrogen cryostat (Cosier & Glazer, 1986) operating at 100.0 (1) K.
Geometry. All e.s.d.'s (except the e.s.d. in the dihedral angle between two l.s. planes) are estimated using the full covariance matrix. The cell e.s.d.'s are taken into account individually in the estimation of e.s.d.'s in distances, angles and torsion angles; correlations between e.s.d.'s in cell parameters are only used when they are defined by crystal symmetry. An approximate (isotropic) treatment of cell e.s.d.'s is used for estimating e.s.d.'s involving l.s. planes.
Refinement. Refinement of *F*^2^ against ALL reflections. The weighted *R*-factor *wR* and goodness of fit *S* are based on *F*^2^, conventional *R*-factors *R* are based on *F*, with *F* set to zero for negative *F*^2^. The threshold expression of *F*^2^ > σ(*F*^2^) is used only for calculating *R*-factors(gt) *etc*. and is not relevant to the choice of reflections for refinement. *R*-factors based on *F*^2^ are statistically about twice as large as those based on *F*, and *R*- factors based on ALL data will be even larger.

### Fractional atomic coordinates and isotropic or equivalent isotropic displacement parameters (Å^2^)

**Table:**

	*x*	*y*	*z*	*U*_iso_*/*U*_eq_	
O1	0.32025 (10)	0.62737 (7)	0.08287 (6)	0.02053 (17)	
O2	0.09201 (10)	0.37202 (7)	0.46035 (6)	0.01980 (17)	
N1	0.32443 (12)	0.49196 (8)	0.39689 (7)	0.01640 (18)	
N2	0.02522 (11)	0.43249 (8)	0.30384 (7)	0.01590 (18)	
C1	0.35063 (15)	0.26503 (13)	−0.05556 (10)	0.0295 (3)	
H1A	0.4532	0.2745	−0.0588	0.035*	
C2	0.32456 (17)	0.18284 (14)	−0.10330 (11)	0.0353 (3)	
H2A	0.4097	0.1357	−0.1380	0.042*	
C3	0.17561 (17)	0.16893 (12)	−0.10080 (10)	0.0288 (3)	
H3A	0.1587	0.1134	−0.1345	0.035*	
C4	0.05189 (15)	0.23645 (11)	−0.04897 (9)	0.0239 (2)	
H4A	−0.0504	0.2269	−0.0465	0.029*	
C5	0.07717 (14)	0.31847 (10)	−0.00041 (9)	0.0203 (2)	
H5A	−0.0084	0.3647	0.0348	0.024*	
C6	0.22671 (13)	0.33376 (10)	−0.00282 (8)	0.0187 (2)	
C7	0.25859 (13)	0.42463 (10)	0.04118 (8)	0.0174 (2)	
H7A	0.3464	0.4605	0.0057	0.021*	
C8	0.18105 (12)	0.46436 (9)	0.12463 (8)	0.0155 (2)	
C9	0.24085 (12)	0.56247 (9)	0.14710 (8)	0.01496 (19)	
C10	0.20008 (12)	0.57123 (9)	0.25602 (8)	0.01411 (19)	
C11	0.02732 (12)	0.55944 (9)	0.29624 (8)	0.0160 (2)	
H11A	−0.0112	0.5819	0.3625	0.019*	
H11B	−0.0364	0.6103	0.2493	0.019*	
C12	0.04794 (13)	0.41191 (10)	0.20116 (8)	0.0169 (2)	
H12A	−0.0485	0.4460	0.1767	0.020*	
H12B	0.0657	0.3249	0.2044	0.020*	
C13	0.25497 (12)	0.67230 (9)	0.28182 (8)	0.01462 (19)	
H13A	0.3614	0.6780	0.2410	0.018*	
C14	0.15851 (13)	0.79502 (9)	0.26271 (8)	0.0161 (2)	
C15	0.20500 (14)	0.87610 (10)	0.17463 (9)	0.0213 (2)	
H15A	0.2932	0.8521	0.1267	0.026*	
C16	0.12390 (16)	0.99138 (11)	0.15618 (10)	0.0270 (3)	
H16A	0.1577	1.0456	0.0962	0.032*	
C17	−0.00561 (15)	1.02738 (11)	0.22473 (11)	0.0274 (3)	
H17A	−0.0604	1.1063	0.2123	0.033*	
C18	−0.05505 (14)	0.94748 (11)	0.31188 (11)	0.0254 (3)	
H18A	−0.1450	0.9715	0.3586	0.031*	
C19	0.02673 (13)	0.83210 (10)	0.33122 (9)	0.0205 (2)	
H19A	−0.0074	0.7783	0.3914	0.025*	
C20	0.26890 (13)	0.62139 (9)	0.39205 (8)	0.0152 (2)	
H20A	0.1631	0.6314	0.4352	0.018*	
C21	0.36922 (13)	0.68215 (10)	0.42803 (8)	0.0176 (2)	
C22	0.51712 (14)	0.69861 (11)	0.37386 (9)	0.0235 (2)	
H22A	0.5599	0.6654	0.3158	0.028*	
C23	0.60243 (16)	0.76334 (12)	0.40433 (10)	0.0295 (3)	
H23A	0.7029	0.7745	0.3670	0.035*	
C24	0.54059 (18)	0.81159 (12)	0.48937 (11)	0.0322 (3)	
H24A	0.5974	0.8578	0.5090	0.039*	
C25	0.39640 (18)	0.79237 (12)	0.54545 (10)	0.0305 (3)	
H25A	0.3557	0.8233	0.6047	0.037*	
C26	0.31045 (15)	0.72756 (11)	0.51517 (9)	0.0232 (2)	
H26A	0.2115	0.7143	0.5541	0.028*	
C27	0.28286 (12)	0.45467 (9)	0.31644 (8)	0.01400 (19)	
C28	0.15025 (12)	0.37294 (9)	0.35756 (8)	0.0156 (2)	
C29	0.22707 (13)	0.25205 (10)	0.33599 (8)	0.0173 (2)	
C30	0.17969 (15)	0.14266 (10)	0.36601 (9)	0.0221 (2)	
H30A	0.0804	0.1339	0.4065	0.027*	
C31	0.28329 (16)	0.04309 (11)	0.33475 (10)	0.0257 (3)	
H31A	0.2527	−0.0333	0.3565	0.031*	
C32	0.42586 (15)	0.05332 (11)	0.27435 (10)	0.0254 (3)	
H32A	0.4910	−0.0152	0.2540	0.031*	
C33	0.47710 (14)	0.16566 (10)	0.24194 (9)	0.0208 (2)	
C34	0.62171 (14)	0.19104 (12)	0.18143 (10)	0.0253 (3)	
H34A	0.6933	0.1303	0.1529	0.030*	
C35	0.65831 (14)	0.30327 (12)	0.16402 (9)	0.0249 (2)	
H35A	0.7553	0.3183	0.1229	0.030*	
C36	0.55669 (13)	0.39747 (11)	0.20515 (9)	0.0205 (2)	
H36A	0.5866	0.4732	0.1943	0.025*	
C37	0.41441 (12)	0.37650 (10)	0.26087 (8)	0.0158 (2)	
C38	0.37522 (13)	0.26199 (10)	0.27718 (8)	0.0167 (2)	
H1O2	0.167 (2)	0.4027 (18)	0.4756 (16)	0.055 (6)*	
H1N1	0.426 (2)	0.4719 (15)	0.3950 (12)	0.030 (4)*	

### Atomic displacement parameters (Å^2^)

**Table:**

	*U*^11^	*U*^22^	*U*^33^	*U*^12^	*U*^13^	*U*^23^
O1	0.0248 (4)	0.0209 (4)	0.0165 (4)	−0.0088 (3)	−0.0018 (3)	−0.0029 (3)
O2	0.0228 (4)	0.0228 (4)	0.0138 (4)	−0.0077 (3)	0.0000 (3)	−0.0043 (3)
N1	0.0206 (5)	0.0133 (4)	0.0171 (4)	−0.0025 (3)	−0.0067 (4)	−0.0040 (3)
N2	0.0166 (4)	0.0161 (4)	0.0155 (4)	−0.0037 (3)	−0.0019 (3)	−0.0048 (3)
C1	0.0239 (6)	0.0365 (7)	0.0318 (7)	−0.0111 (5)	0.0045 (5)	−0.0193 (6)
C2	0.0338 (7)	0.0395 (8)	0.0362 (7)	−0.0106 (6)	0.0068 (6)	−0.0250 (6)
C3	0.0404 (7)	0.0280 (6)	0.0232 (6)	−0.0129 (6)	−0.0062 (5)	−0.0092 (5)
C4	0.0293 (6)	0.0231 (6)	0.0232 (5)	−0.0078 (5)	−0.0129 (5)	−0.0014 (4)
C5	0.0224 (5)	0.0198 (5)	0.0199 (5)	−0.0027 (4)	−0.0079 (4)	−0.0035 (4)
C6	0.0220 (5)	0.0204 (5)	0.0147 (5)	−0.0060 (4)	−0.0024 (4)	−0.0047 (4)
C7	0.0187 (5)	0.0183 (5)	0.0162 (5)	−0.0051 (4)	−0.0037 (4)	−0.0034 (4)
C8	0.0160 (5)	0.0160 (5)	0.0151 (4)	−0.0032 (4)	−0.0043 (4)	−0.0025 (4)
C9	0.0146 (5)	0.0149 (5)	0.0155 (4)	−0.0016 (4)	−0.0037 (4)	−0.0035 (4)
C10	0.0156 (5)	0.0129 (4)	0.0141 (4)	−0.0027 (4)	−0.0024 (4)	−0.0035 (4)
C11	0.0152 (5)	0.0152 (5)	0.0178 (5)	−0.0022 (4)	−0.0024 (4)	−0.0050 (4)
C12	0.0164 (5)	0.0188 (5)	0.0173 (5)	−0.0051 (4)	−0.0033 (4)	−0.0053 (4)
C13	0.0158 (5)	0.0134 (5)	0.0148 (4)	−0.0030 (4)	−0.0031 (4)	−0.0028 (4)
C14	0.0184 (5)	0.0133 (5)	0.0191 (5)	−0.0037 (4)	−0.0066 (4)	−0.0040 (4)
C15	0.0265 (6)	0.0183 (5)	0.0202 (5)	−0.0049 (4)	−0.0076 (4)	−0.0020 (4)
C16	0.0353 (7)	0.0185 (6)	0.0286 (6)	−0.0060 (5)	−0.0148 (5)	0.0024 (5)
C17	0.0276 (6)	0.0155 (5)	0.0430 (7)	−0.0002 (4)	−0.0184 (6)	−0.0048 (5)
C18	0.0192 (5)	0.0192 (6)	0.0402 (7)	−0.0006 (4)	−0.0076 (5)	−0.0109 (5)
C19	0.0197 (5)	0.0164 (5)	0.0264 (6)	−0.0044 (4)	−0.0042 (4)	−0.0053 (4)
C20	0.0184 (5)	0.0130 (5)	0.0152 (4)	−0.0031 (4)	−0.0039 (4)	−0.0037 (4)
C21	0.0217 (5)	0.0148 (5)	0.0182 (5)	−0.0030 (4)	−0.0080 (4)	−0.0026 (4)
C22	0.0244 (6)	0.0255 (6)	0.0230 (5)	−0.0081 (5)	−0.0075 (5)	−0.0029 (4)
C23	0.0311 (7)	0.0291 (6)	0.0327 (7)	−0.0148 (5)	−0.0165 (5)	0.0045 (5)
C24	0.0472 (8)	0.0214 (6)	0.0378 (7)	−0.0119 (6)	−0.0280 (6)	0.0008 (5)
C25	0.0457 (8)	0.0241 (6)	0.0296 (6)	−0.0003 (6)	−0.0210 (6)	−0.0110 (5)
C26	0.0286 (6)	0.0222 (6)	0.0216 (5)	−0.0015 (5)	−0.0096 (5)	−0.0074 (4)
C27	0.0155 (5)	0.0130 (4)	0.0138 (4)	−0.0028 (4)	−0.0025 (4)	−0.0034 (3)
C28	0.0174 (5)	0.0149 (5)	0.0143 (4)	−0.0040 (4)	−0.0016 (4)	−0.0035 (4)
C29	0.0205 (5)	0.0152 (5)	0.0179 (5)	−0.0029 (4)	−0.0058 (4)	−0.0045 (4)
C30	0.0263 (6)	0.0176 (5)	0.0250 (6)	−0.0067 (4)	−0.0086 (5)	−0.0031 (4)
C31	0.0343 (7)	0.0153 (5)	0.0326 (6)	−0.0048 (5)	−0.0149 (5)	−0.0058 (5)
C32	0.0316 (6)	0.0174 (5)	0.0318 (6)	0.0031 (5)	−0.0143 (5)	−0.0115 (5)
C33	0.0234 (6)	0.0199 (5)	0.0217 (5)	0.0013 (4)	−0.0091 (4)	−0.0088 (4)
C34	0.0217 (6)	0.0282 (6)	0.0259 (6)	0.0051 (5)	−0.0050 (5)	−0.0131 (5)
C35	0.0170 (5)	0.0299 (6)	0.0245 (6)	0.0003 (5)	−0.0003 (4)	−0.0072 (5)
C36	0.0183 (5)	0.0212 (5)	0.0206 (5)	−0.0025 (4)	−0.0032 (4)	−0.0032 (4)
C37	0.0161 (5)	0.0165 (5)	0.0147 (5)	−0.0008 (4)	−0.0038 (4)	−0.0040 (4)
C38	0.0195 (5)	0.0150 (5)	0.0169 (5)	−0.0003 (4)	−0.0067 (4)	−0.0049 (4)

### Geometric parameters (Å, °)

**Table:**

O1—C9	1.2200 (13)	C16—C17	1.382 (2)
O2—C28	1.4095 (13)	C16—H16A	0.9500
O2—H1O2	0.92 (2)	C17—C18	1.3883 (19)
N1—C27	1.4650 (13)	C17—H17A	0.9500
N1—C20	1.4841 (14)	C18—C19	1.3957 (16)
N1—H1N1	0.895 (17)	C18—H18A	0.9500
N2—C11	1.4705 (14)	C19—H19A	0.9500
N2—C28	1.4772 (14)	C20—C21	1.5105 (15)
N2—C12	1.4809 (14)	C20—H20A	1.0000
C1—C2	1.3902 (18)	C21—C26	1.3945 (16)
C1—C6	1.3976 (17)	C21—C22	1.3957 (17)
C1—H1A	0.9500	C22—C23	1.3926 (17)
C2—C3	1.388 (2)	C22—H22A	0.9500
C2—H2A	0.9500	C23—C24	1.389 (2)
C3—C4	1.3830 (19)	C23—H23A	0.9500
C3—H3A	0.9500	C24—C25	1.382 (2)
C4—C5	1.3936 (16)	C24—H24A	0.9500
C4—H4A	0.9500	C25—C26	1.3960 (17)
C5—C6	1.3984 (16)	C25—H25A	0.9500
C5—H5A	0.9500	C26—H26A	0.9500
C6—C7	1.4662 (15)	C27—C37	1.5145 (15)
C7—C8	1.3443 (15)	C27—C28	1.6054 (15)
C7—H7A	0.9500	C28—C29	1.5069 (15)
C8—C9	1.5006 (15)	C29—C30	1.3747 (16)
C8—C12	1.5302 (15)	C29—C38	1.4037 (16)
C9—C10	1.5091 (14)	C30—C31	1.4200 (17)
C10—C13	1.5300 (14)	C30—H30A	0.9500
C10—C11	1.5524 (15)	C31—C32	1.3705 (19)
C10—C27	1.5715 (15)	C31—H31A	0.9500
C11—H11A	0.9900	C32—C33	1.4222 (17)
C11—H11B	0.9900	C32—H32A	0.9500
C12—H12A	0.9900	C33—C38	1.4056 (15)
C12—H12B	0.9900	C33—C34	1.4217 (18)
C13—C14	1.5167 (15)	C34—C35	1.3756 (19)
C13—C20	1.5478 (15)	C34—H34A	0.9500
C13—H13A	1.0000	C35—C36	1.4201 (17)
C14—C15	1.3977 (16)	C35—H35A	0.9500
C14—C19	1.3984 (16)	C36—C37	1.3707 (15)
C15—C16	1.3914 (17)	C36—H36A	0.9500
C15—H15A	0.9500	C37—C38	1.4128 (15)
			
C28—O2—H1O2	101.1 (13)	C17—C18—H18A	119.8
C27—N1—C20	109.79 (8)	C19—C18—H18A	119.8
C27—N1—H1N1	111.1 (11)	C18—C19—C14	120.53 (11)
C20—N1—H1N1	112.7 (10)	C18—C19—H19A	119.7
C11—N2—C28	102.58 (8)	C14—C19—H19A	119.7
C11—N2—C12	107.90 (8)	N1—C20—C21	113.79 (9)
C28—N2—C12	115.78 (9)	N1—C20—C13	104.68 (8)
C2—C1—C6	120.34 (12)	C21—C20—C13	113.90 (9)
C2—C1—H1A	119.8	N1—C20—H20A	108.1
C6—C1—H1A	119.8	C21—C20—H20A	108.1
C3—C2—C1	120.74 (13)	C13—C20—H20A	108.1
C3—C2—H2A	119.6	C26—C21—C22	119.05 (11)
C1—C2—H2A	119.6	C26—C21—C20	119.30 (10)
C4—C3—C2	119.54 (12)	C22—C21—C20	121.59 (10)
C4—C3—H3A	120.2	C23—C22—C21	120.44 (12)
C2—C3—H3A	120.2	C23—C22—H22A	119.8
C3—C4—C5	120.03 (12)	C21—C22—H22A	119.8
C3—C4—H4A	120.0	C24—C23—C22	119.96 (13)
C5—C4—H4A	120.0	C24—C23—H23A	120.0
C4—C5—C6	120.97 (11)	C22—C23—H23A	120.0
C4—C5—H5A	119.5	C25—C24—C23	120.04 (12)
C6—C5—H5A	119.5	C25—C24—H24A	120.0
C1—C6—C5	118.38 (11)	C23—C24—H24A	120.0
C1—C6—C7	118.64 (10)	C24—C25—C26	120.16 (12)
C5—C6—C7	122.82 (10)	C24—C25—H25A	119.9
C8—C7—C6	129.09 (10)	C26—C25—H25A	119.9
C8—C7—H7A	115.5	C21—C26—C25	120.27 (12)
C6—C7—H7A	115.5	C21—C26—H26A	119.9
C7—C8—C9	115.80 (10)	C25—C26—H26A	119.9
C7—C8—C12	125.78 (10)	N1—C27—C37	113.15 (9)
C9—C8—C12	118.22 (9)	N1—C27—C10	105.52 (8)
O1—C9—C8	122.57 (10)	C37—C27—C10	119.35 (8)
O1—C9—C10	122.77 (9)	N1—C27—C28	112.48 (8)
C8—C9—C10	114.62 (9)	C37—C27—C28	103.27 (8)
C9—C10—C13	116.33 (9)	C10—C27—C28	102.71 (8)
C9—C10—C11	107.84 (8)	O2—C28—N2	107.94 (9)
C13—C10—C11	116.86 (9)	O2—C28—C29	113.38 (9)
C9—C10—C27	107.93 (8)	N2—C28—C29	114.94 (9)
C13—C10—C27	104.25 (8)	O2—C28—C27	109.13 (8)
C11—C10—C27	102.26 (8)	N2—C28—C27	105.97 (8)
N2—C11—C10	103.74 (8)	C29—C28—C27	105.07 (8)
N2—C11—H11A	111.0	C30—C29—C38	119.65 (11)
C10—C11—H11A	111.0	C30—C29—C28	131.97 (11)
N2—C11—H11B	111.0	C38—C29—C28	108.33 (9)
C10—C11—H11B	111.0	C29—C30—C31	118.17 (11)
H11A—C11—H11B	109.0	C29—C30—H30A	120.9
N2—C12—C8	115.52 (9)	C31—C30—H30A	120.9
N2—C12—H12A	108.4	C32—C31—C30	122.28 (11)
C8—C12—H12A	108.4	C32—C31—H31A	118.9
N2—C12—H12B	108.4	C30—C31—H31A	118.9
C8—C12—H12B	108.4	C31—C32—C33	120.55 (11)
H12A—C12—H12B	107.5	C31—C32—H32A	119.7
C14—C13—C10	116.62 (9)	C33—C32—H32A	119.7
C14—C13—C20	115.23 (9)	C38—C33—C34	116.17 (11)
C10—C13—C20	101.96 (8)	C38—C33—C32	116.15 (11)
C14—C13—H13A	107.5	C34—C33—C32	127.63 (11)
C10—C13—H13A	107.5	C35—C34—C33	120.22 (11)
C20—C13—H13A	107.5	C35—C34—H34A	119.9
C15—C14—C19	118.28 (10)	C33—C34—H34A	119.9
C15—C14—C13	119.04 (10)	C34—C35—C36	122.51 (11)
C19—C14—C13	122.65 (10)	C34—C35—H35A	118.7
C16—C15—C14	120.96 (12)	C36—C35—H35A	118.7
C16—C15—H15A	119.5	C37—C36—C35	118.40 (11)
C14—C15—H15A	119.5	C37—C36—H36A	120.8
C17—C16—C15	120.29 (12)	C35—C36—H36A	120.8
C17—C16—H16A	119.9	C36—C37—C38	119.20 (10)
C15—C16—H16A	119.9	C36—C37—C27	131.72 (10)
C16—C17—C18	119.58 (11)	C38—C37—C27	109.02 (9)
C16—C17—H17A	120.2	C29—C38—C33	123.08 (10)
C18—C17—H17A	120.2	C29—C38—C37	113.51 (10)
C17—C18—C19	120.36 (12)	C33—C38—C37	123.35 (11)
			
C6—C1—C2—C3	1.0 (2)	C20—C21—C26—C25	−174.86 (11)
C1—C2—C3—C4	−0.9 (2)	C24—C25—C26—C21	−0.22 (19)
C2—C3—C4—C5	0.5 (2)	C20—N1—C27—C37	134.03 (9)
C3—C4—C5—C6	−0.20 (18)	C20—N1—C27—C10	1.81 (11)
C2—C1—C6—C5	−0.7 (2)	C20—N1—C27—C28	−109.42 (10)
C2—C1—C6—C7	−176.23 (13)	C9—C10—C27—N1	145.40 (9)
C4—C5—C6—C1	0.26 (18)	C13—C10—C27—N1	21.12 (10)
C4—C5—C6—C7	175.63 (11)	C11—C10—C27—N1	−101.03 (9)
C1—C6—C7—C8	−148.67 (13)	C9—C10—C27—C37	16.77 (12)
C5—C6—C7—C8	35.97 (18)	C13—C10—C27—C37	−107.51 (10)
C6—C7—C8—C9	−177.69 (11)	C11—C10—C27—C37	130.34 (9)
C6—C7—C8—C12	7.47 (19)	C9—C10—C27—C28	−96.60 (9)
C7—C8—C9—O1	21.80 (15)	C13—C10—C27—C28	139.12 (8)
C12—C8—C9—O1	−162.96 (10)	C11—C10—C27—C28	16.97 (9)
C7—C8—C9—C10	−155.94 (10)	C11—N2—C28—O2	79.87 (9)
C12—C8—C9—C10	19.31 (13)	C12—N2—C28—O2	−162.90 (8)
O1—C9—C10—C13	3.92 (15)	C11—N2—C28—C29	−152.50 (9)
C8—C9—C10—C13	−178.35 (9)	C12—N2—C28—C29	−35.27 (13)
O1—C9—C10—C11	137.47 (11)	C11—N2—C28—C27	−36.94 (10)
C8—C9—C10—C11	−44.80 (11)	C12—N2—C28—C27	80.29 (10)
O1—C9—C10—C27	−112.75 (11)	N1—C27—C28—O2	8.33 (12)
C8—C9—C10—C27	64.98 (11)	C37—C27—C28—O2	130.65 (9)
C28—N2—C11—C10	48.78 (10)	C10—C27—C28—O2	−104.64 (9)
C12—N2—C11—C10	−73.93 (10)	N1—C27—C28—N2	124.34 (9)
C9—C10—C11—N2	73.02 (10)	C37—C27—C28—N2	−113.34 (9)
C13—C10—C11—N2	−153.71 (9)	C10—C27—C28—N2	11.37 (10)
C27—C10—C11—N2	−40.61 (10)	N1—C27—C28—C29	−113.57 (9)
C11—N2—C12—C8	47.97 (12)	C37—C27—C28—C29	8.76 (10)
C28—N2—C12—C8	−66.24 (12)	C10—C27—C28—C29	133.47 (8)
C7—C8—C12—N2	154.71 (11)	O2—C28—C29—C30	50.25 (16)
C9—C8—C12—N2	−20.01 (14)	N2—C28—C29—C30	−74.59 (15)
C9—C10—C13—C14	80.35 (12)	C27—C28—C29—C30	169.34 (12)
C11—C10—C13—C14	−48.99 (13)	O2—C28—C29—C38	−127.09 (10)
C27—C10—C13—C14	−160.96 (9)	N2—C28—C29—C38	108.08 (10)
C9—C10—C13—C20	−153.24 (9)	C27—C28—C29—C38	−8.00 (11)
C11—C10—C13—C20	77.42 (10)	C38—C29—C30—C31	−0.81 (17)
C27—C10—C13—C20	−34.55 (10)	C28—C29—C30—C31	−177.90 (11)
C10—C13—C14—C15	−98.20 (12)	C29—C30—C31—C32	−1.57 (18)
C20—C13—C14—C15	142.30 (10)	C30—C31—C32—C33	1.29 (19)
C10—C13—C14—C19	83.78 (13)	C31—C32—C33—C38	1.32 (17)
C20—C13—C14—C19	−35.72 (14)	C31—C32—C33—C34	178.55 (12)
C19—C14—C15—C16	1.04 (17)	C38—C33—C34—C35	2.95 (17)
C13—C14—C15—C16	−177.07 (10)	C32—C33—C34—C35	−174.28 (12)
C14—C15—C16—C17	−0.61 (18)	C33—C34—C35—C36	0.48 (19)
C15—C16—C17—C18	−0.47 (19)	C34—C35—C36—C37	−2.72 (18)
C16—C17—C18—C19	1.10 (19)	C35—C36—C37—C38	1.34 (16)
C17—C18—C19—C14	−0.66 (18)	C35—C36—C37—C27	178.17 (11)
C15—C14—C19—C18	−0.41 (17)	N1—C27—C37—C36	−61.88 (15)
C13—C14—C19—C18	177.63 (10)	C10—C27—C37—C36	63.17 (15)
C27—N1—C20—C21	−148.81 (9)	C28—C27—C37—C36	176.24 (11)
C27—N1—C20—C13	−23.83 (11)	N1—C27—C37—C38	115.19 (10)
C14—C13—C20—N1	163.34 (9)	C10—C27—C37—C38	−119.76 (10)
C10—C13—C20—N1	36.02 (10)	C28—C27—C37—C38	−6.69 (11)
C14—C13—C20—C21	−71.76 (12)	C30—C29—C38—C33	3.60 (17)
C10—C13—C20—C21	160.93 (9)	C28—C29—C38—C33	−178.68 (10)
N1—C20—C21—C26	−113.39 (11)	C30—C29—C38—C37	−173.61 (10)
C13—C20—C21—C26	126.72 (11)	C28—C29—C38—C37	4.10 (13)
N1—C20—C21—C22	69.44 (13)	C34—C33—C38—C29	178.66 (10)
C13—C20—C21—C22	−50.46 (14)	C32—C33—C38—C29	−3.78 (16)
C26—C21—C22—C23	−2.41 (17)	C34—C33—C38—C37	−4.40 (16)
C20—C21—C22—C23	174.77 (11)	C32—C33—C38—C37	173.16 (10)
C21—C22—C23—C24	0.25 (19)	C36—C37—C38—C29	179.49 (10)
C22—C23—C24—C25	1.96 (19)	C27—C37—C38—C29	1.99 (13)
C23—C24—C25—C26	−1.98 (19)	C36—C37—C38—C33	2.29 (16)
C22—C21—C26—C25	2.39 (17)	C27—C37—C38—C33	−175.21 (10)

### Hydrogen-bond geometry (Å, °)

**Table:**

*D*—H···*A*	*D*—H	H···*A*	*D*···*A*	*D*—H···*A*
O2—H1O2···N1	0.93 (2)	1.91 (2)	2.6348 (14)	133.7 (18)
C1—H1A···O1^i^	0.95	2.48	3.3874 (17)	160
C11—H11A···O2^ii^	0.99	2.57	3.5621 (13)	175
C19—H19A···O2^ii^	0.95	2.46	3.4044 (14)	176
C20—H20A···O2^ii^	1.00	2.42	3.4090 (15)	172

Symmetry codes: (i) −*x*+1, −*y*+1, −*z*; (ii) −*x*, −*y*+1, −*z*+1.
